# Liver Graft Susceptibility during Static Cold Storage and Dynamic Machine Perfusion: DCD versus Fatty Livers

**DOI:** 10.3390/ijms19010109

**Published:** 2017-12-31

**Authors:** Andrea Ferrigno, Laura G. Di Pasqua, Clarissa Berardo, Veronica Siciliano, Vittoria Rizzo, Barbara Mannucci, Plinio Richelmi, Anna Cleta Croce, Mariapia Vairetti

**Affiliations:** 1 Department of Internal Medicine and Therapeutics, Unit of Cellular and Molecular Pharmacology and Toxicology, University of Pavia, 27100 Pavia, Italy; andrea.ferrigno@unipv.it (A.F.); lauragiuseppin.dipasqua01@universitadipavia.it (L.G.D.P.); clarissa.berardo01@universitadipavia.it (C.B.); veronica.siciliano01@universitadipavia.it (V.S.); plinio.richelmi@unipv.it (P.R.); 2 Department of Molecular Science, IRCCS S. Matteo, University of Pavia, 27100 Pavia, Italy; v.rizzo@smatteo.pv.it; 3 Centro Grandi Strumenti, University of Pavia, 27100 Pavia, Italy; barbara.mannucci@unipv.it; 4 Institute of Molecular Genetics, Italian National Research Council (CNR), 27100 Pavia, Italy; leta@igm.cnr.it; 5 Department of Biology and Biotechnology, University of Pavia, 27100 Pavia, Italy

**Keywords:** marginal livers, preservation, static cold storage, dynamic machine perfusion, DCD livers, fatty livers

## Abstract

We compared static preservation (cold storage, CS, 4 °C) with dynamic preservation (machine perfusion, MP, 20 °C) followed by reperfusion using marginal livers: a model of donation after cardiac death (DCD) livers and two models of fatty livers, the methionine-choline deficient (MCD) diet model, and obese Zucker (fa/fa) rats. CS injury in DCD livers was reversed by an oxygenated washout (OW): hepatic damage, bile flow, and the ATP/ADP ratio in the OW + CS group was comparable with the ratio obtained with MP. Using fatty livers, CS preservation induced a marked release in hepatic and biliary enzymes in obese Zucker rats when compared with the MCD group. The same trend occurred for bile flow. No difference was found when comparing MP in MCD and obese Zucker rats. Fatty acid analysis demonstrated that the total saturated (SFA)/polyunsaturated fatty acid (PUFA) ratio was, respectively, 1.5 and 0.71 in obese Zucker and MCD rats. While preservation damage in DCD livers is associated with the ATP/ADP recovered with OW, injury in fatty livers is linked to fatty acid constituents: livers from obese. Zucker rats, with greater content in saturated FA, might be more prone to CS injury. On the contrary, MCD livers with elevated PUFA content might be less susceptible to hypothermia.

## 1. Introduction

Since potential recipients on waiting lists persistently outnumber donors, in the last few years there has been renewed interest in both donation after cardiac death (DCD) liver and the use of fatty livers as a potential method of increasing the pool of organs available. DCD or fatty livers are defined as marginal organs with an increased risk for graft dysfunction, primary non-function, and biliary complications that may subject the recipient to greater risks of morbidity or mortality [1]. Multiple methods are currently being investigated to allow the use of marginal organs by minimizing the effects of ischemia/reperfusion (I/R) injury occurring during transplantation [2]. Dynamic machine perfusion (MP) is a technique used in organ transplantation as a means of preserving the organs to be transplanted and is an alternative to static cold storage (CS) [3]. Although conventional hypothermic CS remains the primary method for liver preservation, largely because of its cost-effectiveness, simplicity and logistics, several studies have reported results regarding the use of dynamic MP performed under hypothermic or subnormothermic or normothermic conditions, as an innovative approach to reclaiming marginal livers [4,5].

Following on from results obtained from experimental models, hypothermic MP has been used in patients by Guarrera et al. who demonstrated that, when using livers from DCD donors, MP decreases the extent of graft injury, improves allograft function, lowers serum transaminases, and decreases hospital stays as compared with liver preserved by static CS [6,7]. Other studies have reported that hypothermic oxygenated perfusion (HOPE) was successfully applied for 1 to 2 h prior to implantation: in a trial using human DCD livers the use of HOPE decreased intrahepatic cholangiopathy and biliary complications and improved one-year graft survival [8,9]. In 2009, we published the first paper on the use of subnormothermic MP performed at 20 °C in rat livers with steatosis: the enzymes released in steatotic livers preserved by MP at 20 °C were similar to those shown in non-steatotic organs. Bile production and the ATP/ADP ratio were higher while biliary enzymes and oxidative stress were reduced in fatty livers preserved with MP 20 °C versus those preserved with CS [10]. We further demonstrated that in both rat and swine models MP at 20 °C could be also used in DCD livers [11,12]. Furthermore, using an orthotopic transplantation model, subnormothermic MP improved ischemic damage in livers [13]. Human livers preserved by subnormothermic MP were metabolically active and a significant improvement in bile production and tissue ATP content were found [14]. The advantages of normothermic MP have also been demonstrated both in DCD and steatotic livers. An updated list of experimental and clinical studies are reported in the review by Kollmann and Selzner [5].

Although some studies have reported the clinical use of dynamic MP preservation in humans, current clinical practice regarding liver preservation is based on conventional static CS. On the other hand, there is an urgent need to explore new strategies that provide a more efficient preservation of marginal liver grafts. In this study, we compared preservation injury using static CS and dynamic MP at 20 °C in DCD livers and in two models of non-alcoholic fatty liver disease (NAFLD): MCD diet and obese Zucker fa/fa rats. Since a primary factor limiting the use of marginal livers relates to concerns over the subsequent development of ischemic-type biliary lesions, bile flow and biliary enzyme release were taken into consideration. In addition, lipidomic liver profiling, as a sensitive indicator of graft function, is presented below and discussed comparing CS versus MP preservation injury in marginal livers.

## 2. Results

### 2.1. Effects of Static CS and Dynamic MP Preservation Using DCD Livers

**Figure 1:**
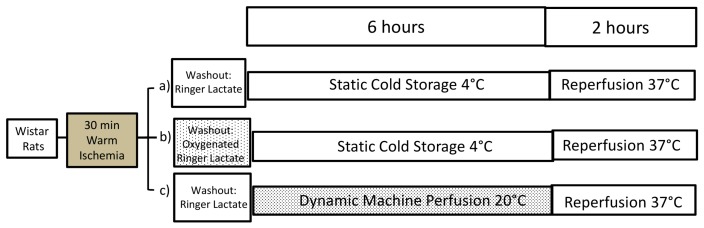
The experimental protocol we adopted using livers from DCD Wistar rats. In the control group (**a**) the DCD graft was preserved with University of Wisconsin (UW) solution at 4 °C (Cold Storage, CS). In group (**b**) an oxygenated washout was performed before CS preservation. In group (**c**) the livers were preserved by machine perfusion preservation at 20 °C (*n* = 7/group).

We previously documented the advantage of MP at 20 °C versus conventional CS in DCD livers [11], as previous results had suggested the beneficial effects of oxygenated perfusion before CS in DCD livers [15]. In this study, we compared the addition of an OW, using Ringer Lactate, before static CS, not just with simple CS, but also with preservation by dynamic MP using livers retrieved from DCD rats (Figure 1).

**Figure 2:**
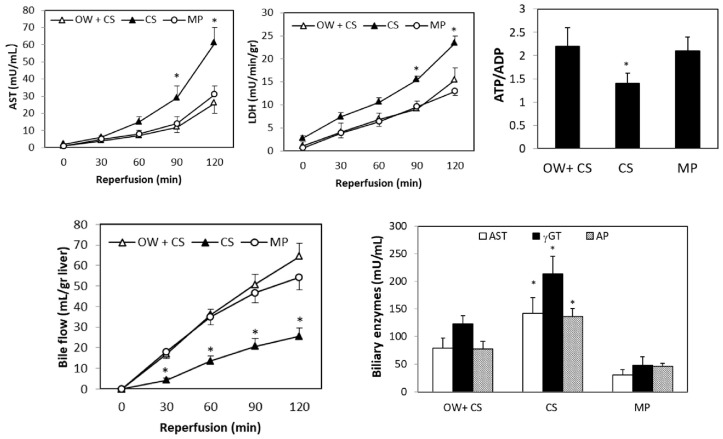
Effects of an oxygenated washout (OW) on enzyme release, ATP/ADP tissue ratio, bile flow and biliary enzymes: alanine-aminotransferase (AST); gamma-glutamyltranferase (γGT); alkaline phosphatase (AP) at the end of reperfusion. After 30 min ischemia, livers were submitted to OW + CS or CS or MP; after 6 h preservation, 2 h reperfusion was performed. The values are reported as means ± standard error (SE), *n* = 7/group. * <0.05 versus OW + CS or MP.

OW significantly reduced hepatic enzyme release in DCD livers and increased both bile flow and the ATP/ADP tissue ratio when compared with organs submitted to simple CS (Figure 2). Moreover, biliary enzymes were lower in OW + CS livers as compared with the CS group (Figure 2). Total bile production was 56 ± 7 versus 26 ± 4 µL/g liver, in the OW + CS and CS groups, respectively. There was no difference in hepatic enzyme release or the ATP/ADP ratio was detected when comparing DCD livers preserved by OW + CS or by MP. The same trend was also found for bile flow and biliary enzymes (Figure 2). At the end of reperfusion, total bile production was, respectively, 56 ± 7 versus 54 ± 4 in livers preserved by OW + CS or by MP.

**Figure 3:**
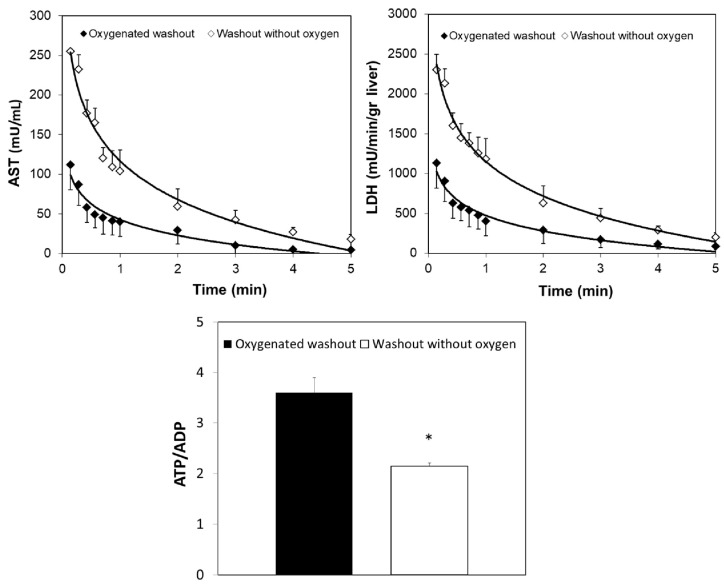
Effects of OW on enzyme release and the ATP/ADP tissue ratio using livers after 30 min ischemia. During the first 5 min of reperfusion, alanine-aminotransferase (AST) and lactate dehydrogenase (LDH) release were measured in the CS livers after washout in the presence or absence of oxygen. The hepatic ATP/ADP ratio was measured at the end of washout before CS preservation. The values are reported as means ± SE, *n =* 7/group. * <0.03.

To clarify the effects of OW, hepatic enzyme release and energy content were also measured in DCD livers at the end of the OW period; the ATP/ADP ratio was higher and enzyme release was lower in DCD organs at the end of OW when compared with liver submitted to washout without oxygen supplementation (Figure 3). At the end of reperfusion, no difference in portal pressure among livers preserved by CS, OW + CS, or MP was observed.

### 2.2. Effects of Static and Dynamic Preservation Using Fatty Livers

**Figure 4:**
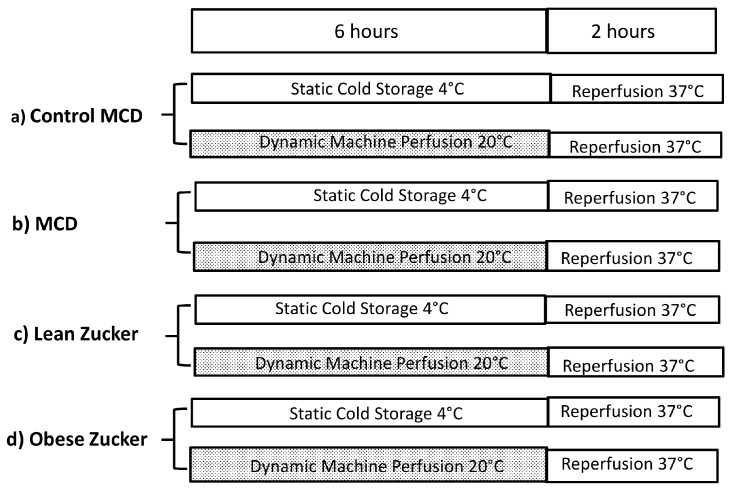
The overall experimental protocol we adopted using fatty livers and their respective controls. Wistar rats were fed with either MCD diet for two weeks (**b**) or control isocaloric diet added with methionine and choline (**a**); obese Zucker rats 11–12 weeks old (fa/fa) (**d**) and lean male Zucker rats (**c**) were used (*n =* 7/group).

We compared static CS and dynamic MP preservation using fatty livers from two week MCD treated rats and ten week obese Zucker rats as summarized in Figure 4. Before organ isolation, these two experimental models exhibited similar concentrations of hepatic serum enzymes suggesting comparable liver injury (Table 1).

**Figure 5:**
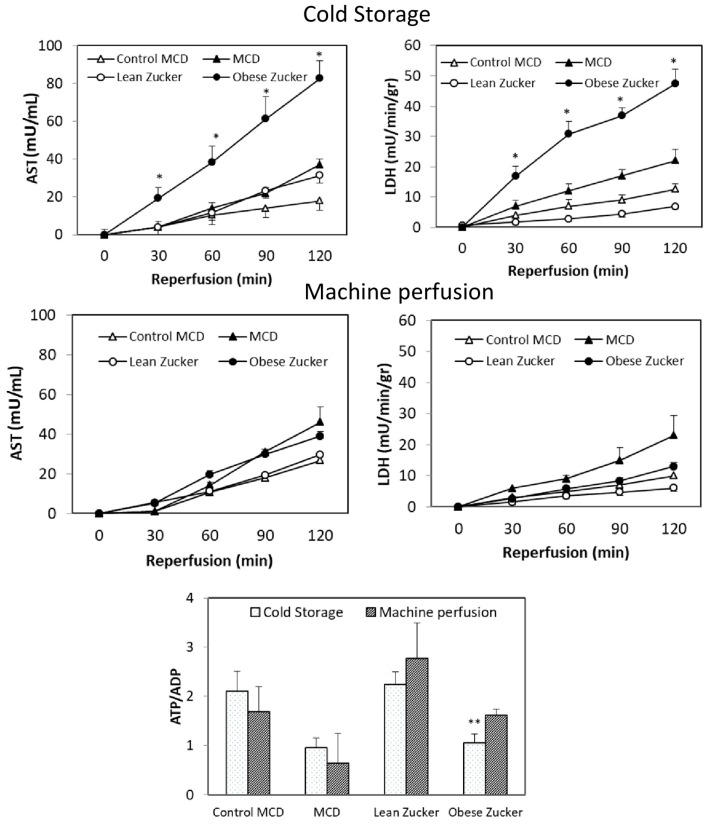
Effects of static CS or dynamic MP in MCD and Zucker rats. AST and LDH release and the ATP/ADP tissue ratio at the end of reperfusion. Livers were submitted to CS or MP; after 6 h preservation, 2 h reperfusion was performed. The values are reported as means ± SE, *n =* 7/group. * <0.05 versus MCD; ** <0.05 versus obese Zucker preserved by MP.

After 6 h CS and 2 h reperfusion, a three-fold increase in AST and a two-fold increase in LDH were found in obese Zucker rats as compared with the MCD group (Figure 5). No difference in tissue ATP/ADP ratio was found in the MCD group comparing with the obese Zucker rats, at the end of reperfusion after CS preservation. Using dynamic MP, a significantly lower hepatic damage as well as an increase in bile flow and in the ATP/ADP ratio were found in livers from obese Zucker rats as compared with static CS preservation (Figure 5). 

**Figure 6:**
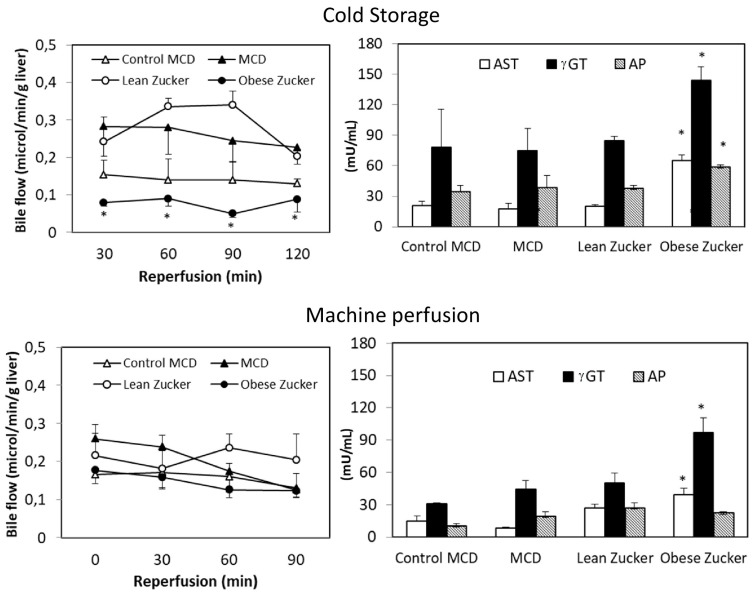
Effects of static CS and dynamic MP on bile flow and biliary enzymes: AST; γGT; AP at the end of reperfusion in MCD and Zucker rats. The values are reported as means ± SE, *n =* 7/group. * <0.05 versus MCD.

Bile flow was higher in the MCD livers preserved by CS than in the obese Zucker group (Figure 6). At the end of reperfusion, total bile production in livers from MCD and Zucker rats was, respectively, 62 ± 13 versus 24 ± 3 µL/g. A similar trend occurred with biliary enzymes: compared with the MCD group, obese Zucker rats had a three-fold increase in AST and a two-fold increase in γ-glutamyltransferase (γGT) (Figure 6).

Using dynamic MP, an increase in bile flow was found in livers from Zucker rats when compared with static CS preservation (Figure 6). No significant difference was detected when we compared MP preservation in MCD with preservation in obese Zucker rats and when we also compared MCD with the control group. A similar trend occurred with hepatic enzyme release, bile flow, and the ATP/ADP ratio (Figure 4 and Figure 5). Biliary levels of AST and γGT were lower in the MCD group when compared with the obese Zucker rats.

The same organ injury and similar ATP/ADP ratios and biliary enzymes were found comparing livers from control MCD and lean Zucker rats submitted to static or dynamic preservation (Figure 4 and Figure 5). Bile flow was higher in lean Zucker rats when compared with the control MCD group (Figure 5). At the end of reperfusion after CS preservation, higher portal pressure was found in obese Zucker rats when compared with the MCD group (mmHg: 24.6 ± 3.4 versus 12.9 ± 1.2). No difference in portal pressure between livers preserved by MP was observed.

### 2.3. Fatty Acid (FA) Constituents

**Figure 7:**
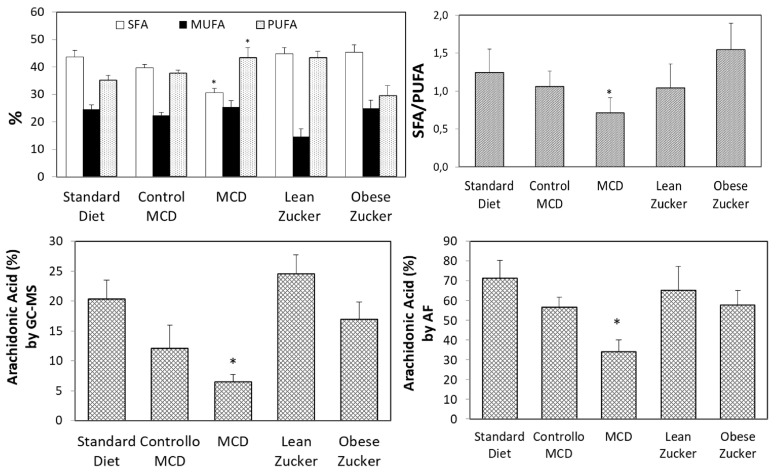
SFA, MUFA, and PUFA measured by GS-MS; arachidonic acid measured by GS-MS and AF in Wistar, MCD and Zucker rats. The values are reported as means ± SE, *n =* 7/group. * <0.05 versus obese Zucker rats.

Assessment of FA constituents with gas chromatography-mass spectrometry (GC/MS) and autofluorescence analysis (AF) was performed in all the experimental models used in this study. GC/MS analysis demonstrated comparable results in livers obtained from rats treated with a standard diet or an isocaloric diet with choline and methionine (Control MCD group). No difference in SFA, monosaturated fatty acid (MUFA), and PUFA was found (Figure 7). A comparable percentage of SFA, a decrease in MUFA and an increase in PUFA were obtained by comparing lean Zucker rats with both the standard diet and the Control MCD diet group (Figure 7). On the contrary, a significant difference was found in SFA and PUFA in obese Zucker and MCD rats, as indicated in Figure 7. The SFA/PUFA ratio was 1.5 and 0.71, respectively. No difference in MUFA was found comparing Zucker and MCD rats (Figure 7). In addition, when compared with obese Zucker rats, MCD rats showed a three-fold decrease in saturated stearic acid (6.7 ± 0.9 versus 19.9 ± 3.1), a six-fold increase in polyunsaturated linoleic acid (31.4 ± 2.9 versus 5.1 ± 1.1), and a three-fold decrease in polyunsaturated arachidonic acid (Figure 7). 

Liver tissue AF is an innovative resource when monitoring fluorescing fatty acids in real-time [16]. Using the same liver lipid extract used for GS-MS, assessment of FA constituents using AF demonstrated that arachidonic acid levels reflected the changes observed by standard GC-MS analysis (Figure 7). 

**Figure 8:**
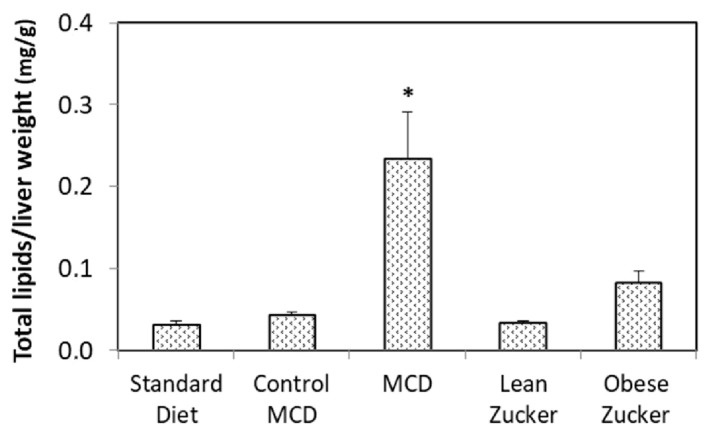
Total lipids in Wistar, MCD, and Zucker rats. The values are means ± SE, *n =* 7/group. * <0.03 versus obese Zucker rats.

Total hepatic lipid content evaluated by Nile Red was higher in MCD rats than in obese Zucker rats (Figure 8). No difference in total lipid content was found when we compared rats treated with a standard diet, rats on a control MCD diet, and lean Zucker rats (Figure 8).

## 3. Discussion

### 3.1. A Comparison between Static versus Dynamic Preservation in DCD and Fatty Livers

Dynamic preservation has emerged as a potential solution to the risk of greater I/R injury in DCD livers, the result of donor warm ischemia time. Several studies have reported that oxygenated perfusion before static CS reduces I/R damage in DCD organs [17,18]. In this study, OW increased the ATP/ADP tissue ratio and reduced liver enzyme release in DCD livers as documented by our analysis before the preservation period. OW also reduced hepatic injury at the end of reperfusion after CS preservation. Liver damage was comparable with what was observed in organs preserved with the dynamic MP technique. The same protection was observed for mitochondrial function as shown by the ATP/ADP ratio. We previously demonstrated that MP preservation at 20 °C improves cellular survival reducing mitochondrial function in livers obtained from DCDs as compared with static preservation by CS using both small and large animal models [11,12]. We also reported previously that injury in DCD livers preserved with MP was similar to what was found using non-ischemic control livers preserved by CS [11]. In the present study, we supported the crucial role of oxygen in DCD organs. Recent results, using end-ischemic dual hypothermic oxygenated machine perfusion (DHOPE) in DCD human liver grafts have demonstrated restoration in hepatic ATP and reduction in reperfusion injury [19]. In the current study, the use of OW before CS reversed liver injury in DCD organs improving the ATP/ADP ratio; the use of MP did not otherwise prevent liver damage.

Fatty livers obtained from both nutritional or genetic models are also particularly susceptible when CS preservation is used [4,20]. We compared fatty livers obtained from two experimental models with comparable hepatic injury. Markedly reduced CS damage was found in MCD livers when compared with organs obtained from obese Zucker rats. The improved level of CS preservation found in MCD livers was not associated with better energy status because the ATP/ADP ratio was similar in both fatty liver models. In addition, total liver lipid content was higher in MCD rats than in obese Zucker rats. On the contrary, obese Zucker rats were particularly prone to static CS injury. Since the most evident difference between Zucker obese rats and MCD rats is the SFA/PUFA ratio, the quality of lipid constituents might influence hepatic injury during preservation by CS more than the quantity of lipid content and energy status. According to this hypothesis, the reduced CS injury observed in MCD livers may be justified by the lower SFA/PUFA ratio, possibly reducing the deleterious tendency to lipid crystallization observed in Zucker obese rats [21]. However, further data need to be collected to demonstrate this hypothesis. 

### 3.2. Ischemic Tract Biliary Lesions: Static versus Dynamic Preservation

Reperfusion of liver grafts after static CS preservation is associated with diminished bile production both in clinical liver transplantation and experimental models. Biliary syndrome is one of the most common complications after liver transplantation [22]. Cholangiocytes play a substantial role in the damage caused by preservation in hypothermic conditions. These cells are particularly susceptible to injury induced by cold hypoxia when compared with parenchymal cells [23]. We have recently shown that hypoxia-inducible factor (HIF)-1α mRNA expression increases in rat livers undergoing cold ischemia [24]. HIF-1α and HIF-2α are important modulators of inflammation [25], showing a pivotal role in the pathogenesis of several forms of liver disease [26]. In particular, cholangiocytes have been shown to modulate the inflammatory secretory profile through upregulation of HIF-1α and HIF-2α, with deleterious consequences on biliary damage [27]. Previous studies have shown that a storage time of more than 10–12 h leads to biliary strictures and other complications in more than 25% of liver transplant recipients [28,29]. Clinical retrospective studies showed a higher re-transplantation rate due to ischemic tract biliary lesions related to serious intrahepatic cholestasis in DCD liver transplantation [24,25,26]. A reduction in post-transplant biliary complications was detected in a clinical study using DHOPE in DCD liver transplantation [19]. The present study underscores the possibility of reducing biliary syndrome in DCD livers with OW before CS. It is not just the use of MP at 20 °C that represents a better chance of preserving the biliary tree. OW before CS also markedly increases bile production and reduces biliary enzymes to an extent that is comparable to what is observed in MP preservation. We previously demonstrated that results with MP preservation were similar to results with non-ischemic livers preserved by CS [11]. What we have reported here is that results with OW + CS are also similar. Thus, the addition of a simple OW appears to be a promising strategy as regards significantly preventing ischemic-type biliary lesions (ITBL) [30].

There is also evidence that post-transplant ITBL occurs in the presence of lipid accumulation, such as in steatosis [31]. Since the recovery of the biliary tree from preservation injury is longer than with hepatocytes or endothelial cells, rapid resumption of biliary secretion is an important index of hepatic functional restoration after preservation [27,28,32,33]. In the present study, we found that, after CS preservation, marked reduction in bile secretion occurred only in obese Zucker rats, but not in fatty livers obtained from MCD rats.

We are aware of the limitations of the rat model of liver preservation injury. However, it also needs to be kept in mind that animal models are essential when it comes to comparing different preservation techniques, such as static CS and dynamic MP, and exploring the mechanisms underlying graft injury. Including marginal livers is strictly connected with the use of innovative technologies that minimize the adverse effects of preservation, decrease reperfusion injury, which ensures better graft survival after transplantation. Recently, Gilbo and Monbaliu, reviewing the role of temperature and oxygenation during dynamic MP, suggested specific perfusion protocols to match different quality grafts [34]. Similarly, the present study suggests that distinct and well-defined perfusion protocols should be used for marginal livers. A modified CS protocol could be used for DCD organs in which preservation damage is strongly associated with hepatic ATP/ADP ratios; CS or MP protocols should be considered in relation to hepatic fatty acid constituents. Fatty livers from obese Zucker donors, characterized by higher levels of saturated FA, are more prone to CS injury as compared with MCD livers with a high PUFA content. On the contrary, livers from MCD rats appear far less susceptible to CS damage, as demonstrated by lower levels of hepatic enzyme release together with decreased biliary damage and increased bile flow. In particular, the very low levels of arachidonic acid found in the MCD group may suggest that a low content in its principal metabolite, 20-hydroxyeicosatetraenoic acid (20-HETE), also occurs in liver from MCD rats. Interestingly 20-HETE was found to be a potent vasoconstrictor of cerebral microvessels, which contributes to I/R injury [35,36]. The role of 20-HETE in the liver is still to be determined even though it represents 50–75% of arachidonic acid metabolites [37]. Previous results in renal transplant patients have demonstrated that extensive 20-HETE release is a negative predictor of post-transplant allograft function [38,39].

## 4. Materials and Methods

### 4.1. Experimental Animals

The animal model used was approved by the Italian Ministry of Health and the Pavia University Animal Care Commission (Document number 2/2012, 25 February, 2012). In the first part, male Wistar rats (eight weeks old from Harlan-Nossan, Correzzana, Italy) were fed with a standard diet. In the second part, male Wistar rats (eight weeks old from Harlan-Nossan) were fed with either a methionine-choline deficient diet, obtained from Piccioni (Roma, Italy), for two weeks (*n =* 14) or a isocolaric control diet (*n =* 14). Obese (fa/fa) and lean (fa/-) male Zucker rats 11–12 week old (Charles River, Calco, Italy) were used as liver donors. The animals were allowed free access to water and food in all the experiments. All the protocols are summarized in Figure 1 and Figure 4.

### 4.2. Experimental Design

In the first part, all the livers were submitted to 30 min warm ischemia [11] and divided into three groups: livers submitted to CS, livers submitted to oxygenated washout before CS and livers submitted to MP at 20 °C (Figure 1).

In the second part we used two models of fatty liver, methionine- and choline-deficient (MCD) diet and obese Zucker fa/fa rats; the isolated livers were submitted to CS or MP at 20 °C (Figure 4).

### 4.3. Preservation Technique and Reperfusion

Static preservation: after washout with Ringer Lactate (50 mL), livers submitted to CS were flushed in situ with UW for 2 min. and maintained at 4 °C in this solution for 6 h [40]. We used UW solution as it is considered the gold standard for liver preservation before transplantation. As reported in the Group 1 protocol, an oxygenated washout was performed before CS in a group of DCD livers.

Dynamic preservation: livers preserved by MP were placed in an organ chamber and connected to a standard recirculating perfusion system. Livers were perfused with oxygenated Krebs–Heinseleit (KH) medium [40]. The KH solution, collected in a reservoir (200 mL), was re-circulated using a roller pump (Gilson Minipuls-3), oxygenated and maintained at 20 °C using a heat exchanger (Julabo-F12); a constant perfusion flow (3.5–4 mL/min/g) was kept during liver perfusion. Using a glass oxygenator the perfusion solution was oxygenated giving a PO_2_ of about 700 mBar at 20 °C. Into the liver chamber the perfusate ran via the suprahepatic caval vein and was re-circulated by the roller pump into the reservoir; air emboli were eliminated from the system with a bubble trap [40].

Reperfusion period with KH (2 h at 37 °C) was performed in the same setup as MP both in CS and MP preserved livers.

Throughout perfusion the portal venous pressure was quantified with a water column connected to the portal vein inflow catheter; before connecting the liver to the circuit, a calibration was performed [41]. The starting perfusion pressure evaluated was about 12–14 mmHg.

### 4.4. Biochemical Determinants

Perfusate samples and hepatic biopsies were collected at the times indicated. Hepatic biopsies from the left lobe were collected and snap frozen in liquid nitrogen.

The release into the effluent perfusate of AST and ALT, evaluated with Hitachi 747 analyzed (Roche/Hitachi, Indianapolis, IN, USA), and LDH, determined as previously reported [42], was used to quantify the hepatocytes’ viability. Total bile production was measured during reperfusion periods and bile flow expressed in µL/min/gr liver [43]; biliary enzymes, γGT, AST, and AP were determined with an automated Hitachi 747 analyzer.

Tissue ATP and ADP was measured with the luciferin-luciferase method, using a bioluminescence assay kit CLS II (Roche Molecular Biochemicals, Milan, Italy) [24].

### 4.5. Fatty Acid Evaluation by GC/MS and AF Analysis

Before preservation, FA profiling of liver tissue was performed on lipid extracts by GS-MS and parallel estimation of fluorescing FA by fitting analysis of AF spectra. Lipid extraction from liver tissue was performed according to Lyn-Cook et al. [44]. Liver samples (50–70 mg each) were homogenized in distilled water, incubated for 1 h with 2:1 chloroform-methanol, and centrifuged to recover the lower lipid phase. This phase was air-dried and resuspended in 100% ethanol. GC/MS analysis was carried out on the derivatized lipid fraction, thus encompassing original free and triglycerides/phospholipid incorporated forms using a ThermoFisher Scientific DSQII system (TraceDSQII mass spectrometer, Trace GC Ultra gas chromatograph, Thermo Fisher Scientific Waltham, MA USA), using Xcalibur MS software version 2.1 (including NIST Mass Spectral Library (NIST 08) and Wiley Registry of Mass Spectral Data 8th Edition for assignment of chemical structures to chromatographic peaks). Each identified peak was expressed as relative percentage areas of total methylated fatty acids (FAME). The reference standard was Marine Oil FAME Mix from Restek (Superchrom S.r.l., Cernusco sul Naviglio, Milan, Italy). Dichloromethane was used as a blank, to avoid carryover from previous analysis. FA are expressed as the relative percentage areas of total fatty acids.

AF spectra were recorded under 366 nm excitation using an LS 55 spectrofluorometer (PerkinElmer Italia, Milan, Italy) equipped with an R 928 photomultiplier detector (PMT, Hamamatsu Photonics, Iwata City, Japan). The fraction (%) of arachidonic acid was calculated from the AF spectra by means of a fitting analysis procedure (PeakFit; SPSS Science, Chicago, IL, USA) based on the Marquardt-Levenberg algorithm [45]. Spectra were normalized to 100 a.u. at the peak maximum, to be processed using half-Gaussian modified Gaussian (GMG) spectral functions representing spectra components, on the basis of the peak center wavelength position (λ) and the full width at the half maximum intensity (FWHM), as already described in [16]. Confidence intervals for the average curve fit were set at 95%, and the quality of fit was verified in accordance with the coefficient of determination (*R*^2^), and residual analysis. FAs evaluated by AF are expressed as relative percentage areas of the total fluorescing fatty acids.

### 4.6. Total Lipid Quantification

To quantify total lipids, aliquots of liver extracts, as described in Section 4.5, were transferred in a 96-well polystyrene white plate, added with phosphate-buffered saline (PBS) and Nile Red (1 mg/mL in DMSO), to measure dye fluorescence intensity (excitation/emission: 485/572 nm; microplate reader, Perkin Elmer Victor X) [46].

## 5. Conclusions

The results of the present study suggest that the choice of preservation strategy varies according to graft quality. Hence specific liver-tailored preservation should be strongly recommended as part of efforts not to discard valuable grafts.

## Figures and Tables

**Table 1:** Serum hepatic enzymes before liver procurement: aspartate aminotransferase (AST), alanine aminotransferase (ALT) and alkaline phosphatase (AP).

Fatty Livers	AST	ALT	AP
MCD rats	89 ± 19	72 ± 11	163 ± 15
Obese Zucker rats	111 ± 12	84 ± 20	181 ± 22

## Abbreviations

20-HETE	20-Hydroxyeicosatetraenoic acid
AF	Autofluorescence
AP	Alkaline phosphatase
ALT	Alanine aminotransferase
AST	Aspartate transaminase
CS	Cold storage
DCD	Donation after cardiac death
DHOPE	Dual hypothermic oxygenated machine perfusion
FA	Fatty acid
γGT	γ-Glutamyltransferase
GS-MS	Gas chromatography–mass spectrometry
HIF	Hypoxia inducible factor
HOPE	Hypothermic oxygenated perfusion
I/R	Ischemia/reperfusion
ITBL	Ischemic-type biliary lesion
LDH	Lactate dehydrogenase
MCD	Methionine choline deficient
MP	Machine perfusion
MUFA	Monoinsatured fatty acid
NAFLD	Non-alcoholic fatty liver disease
OW	Oxygenated washout
PUFA	Polyunsaturated fatty acid
SFA	Saturated fatty acid
